# Social identity bias and communication network clustering interact to shape patterns of opinion dynamics

**DOI:** 10.1098/rsif.2023.0372

**Published:** 2023-12-13

**Authors:** Peter Steiglechner, Paul E. Smaldino, Deyshawn Moser, Agostino Merico

**Affiliations:** ^1^ Systems Ecology Group, Leibniz Centre for Tropical Marine Research (ZMT), Bremen, Germany; ^2^ Institutional and Behavioural Economics Group, Leibniz Centre for Tropical Marine Research (ZMT), Bremen, Germany; ^3^ School of Science, Constructor University, Bremen, Germany; ^4^ School of Business, Social and Decision Sciences, Constructor University, Bremen, Germany; ^5^ Department of Cognitive and Information Sciences, University of California Merced, Merced, CA, USA; ^6^ Santa Fe Institute, Santa Fe, USA

**Keywords:** agent-based model, in-group bias, computational social science, opinion dynamics, generational conflict, climate change

## Abstract

Social influence aligns people's opinions, but social identities and related in-group biases interfere with this alignment. For instance, the recent rise of young climate activists (e.g. ‘Fridays for Future’ or ‘Last Generation’) has highlighted the importance of generational identities in the climate change debate. It is unclear how social identities affect the emergence of opinion patterns, such as consensus or disagreement, in a society. Here, we present an agent-based model to explore this question. Agents communicate in a network and form opinions through social influence. The agents have fixed social identities which involve homophily in their interaction preferences and in-group bias in their perception of others. We find that the in-group bias has opposing effects depending on the network topology. The bias impedes consensus in highly random networks by promoting the formation of echo chambers within social identity groups. By contrast, the bias facilitates consensus in highly clustered networks by aligning dispersed in-group agents across the network and, thereby, preventing the formation of isolated echo chambers. Our model uncovers the mechanisms underpinning these opposing effects of the in-group bias and highlights the importance of the communication network topology for shaping opinion dynamics.

## 1. Introduction

Social influence is a key driver of opinion formation. Opinions should presumably align over time as people discuss an issue and observe what others think about that issue [1–3]. Yet, people do not seem able to find a consensus on many pressing political, environmental or economic issues, including climate change [4], public health measures (like wearing face masks to prevent the spread of COVID-19 [5]) or government-funded social measures [6]. Among these examples, climate change is particularly striking because public opinions remain divided, even though an overwhelming scientific consensus has since long been established. Social influence plays a critical role in this debate [7–10] as pro-environmental attitudes can spread among people, but so can climate skepticism. What makes collective opinion formation a particularly complex problem is that social learning is not a straightforward copying of others' opinions but follows context-dependent strategies [11], which are typically heuristic, affected by cognitive biases [6,12] and based on a limited number of social connections among individuals [13].

A major factor that impacts social influence is social identity [14,15], especially in the debate about climate change [8,16,17]. Individuals affiliate with a specific group, the ‘in-group’, based on similar personal or cultural characteristics such as political orientation, age, sex or occupation. The way they interact with each other depends on whether or not they share such a social identity [18,19]. For example, identity-related factors influenced how people perceived specific policies designed to mitigate the spread of COVID-19 [20]. Similarly, identification with different generations, like ‘youngsters’ or ‘elders’, can add an independent inter-group dimension to the debate on climate change. Age has indeed been shown to correlate with opinions on climate change [21–23]. More importantly, age and related social identity may shape communication and interaction behaviours in the climate change debate by establishing generational in- and out-groups.

Social identity can affect different aspects of communication. First, social identity influences who interacts with whom. People are homophilic, i.e. they preferentially interact with members of their in-group [24,25]. For example, young people observe or discuss more likely the opinions of their young peers and vice versa. Second, social identity influences how individuals perceive socially transmitted information. In particular, people tend to view information coming from in-group sources as more trustworthy and relevant than information originating outside the group. Group membership can thus become an important factor when evaluating the subjective opinions of others. Such differential evaluation of information, known as in-group bias, is a well-studied and persistent feature of human behaviour [16,26–32], although the impacts of this bias vary depending on the culture of the people involved [33] and the nature of the relevant social identities [34]. In-group bias usually reinforces similarities between in-group members [26] and, at the same time, draws like-minded people into the group [16]. Apart from such in-group favouritism, social identity can also involve out-group derogation or aversion, causing divides between groups to become more pronounced [29,34,35]. In summary, social identity does not necessarily determine *what* a particular individual believes about a debated issue like climate change, but social identity can affect (i) *who* that person interacts with and (ii) *how* that person perceives opinions of others.

Mathematical models have become a powerful tool to study opinion formation and constitute a valuable complement to traditional social science approaches, such as laboratory experiments or surveys [9,36]. Models can be used to explore a variety of scenarios, theories or assumptions. In particular, they can provide a link between how cognitive processes, such as in-group bias, manifest at the individual level and how this plays out at the collective level. Most opinion dynamics models are agent-based and simulate how human agents embedded in a social structure update their opinions as they incorporate new information—either through social influence or external stimuli. Such models typically define (i) how people are connected to each other (societal structure), (ii) how their opinions are represented, (iii) how they acquire new information, and (iv) how they process new information to update their opinions (update rule). Homophily or biases are typically integrated by modulating certain components of the model at the individual level. In particular, homophily is often encoded in the societal structure (e.g. [37–39]) and biases are typically encoded in the agents’ update rules (e.g. [40,41]).

Different opinion dynamics models have studied various ways to conceptualize biases (e.g. [38,41,42]). Most of these models focus on biases related exclusively to the exchange of opinions on the debated topic(s). For example, bounded confidence models assume that the opinion distance of two agents fully determines whether they perceive each other as similar and, thus, whether they are influenced by each other. Social identity theory, however, suggests that such biased influence does not depend on people's opinions alone. Perceived similarity and, consequently, the degree of influence between people also depends on their social identities and on group perceptions. We, therefore, argue (in line with previous works [12,15,43–45]) that it is ultimately the interplay of the exchange of opinions *and* of social information that shapes opinion formation. While there is much research in social psychology and related sciences on the influence of social identity on opinion formation—especially at the individual level—such aspects are still understudied with mathematical models of collective opinion formation [43,46,47].

In this study, we investigate the effects of social identity and the related in-group bias on patterns of opinion formation using an agent-based model. Our main assumptions are that individuals align their opinions to those of their social contacts, in line with the social influence literature (e.g. [1,48]), and that social identity moderates such alignment (in line with social identity theory (e.g. [29])), leading to greater shifts in opinions due to interactions from in-group members versus out-group members. Moreover, we assume that individuals interact in non-random ways but that they have stable in- and out-group contacts, which can be represented as a fixed network. While the network topology is in principle uncertain, we assume that agents tend to have more in-group than out-group contacts, in line with literature on homophily (e.g. [25]). In particular, the model is designed along the following principles. Agents hold opinions on a specific topic, for example, climate change, which evolve when they communicate with their neighbors in a fixed network. Agents then use a heuristic approach, formulated following Bayesian calculus, to adapt to perceived opinions. The basic opinion formation process follows previous studies by Martins [49] and Sobkowicz [41]. We extend their framework by including social identity. Agents identify with one of two groups, for example, youngsters or elders in the context of climate change, and we assume that this social identity is visible to others. The effects of social identity are twofold: agents are homophilic in their interaction preferences with respect to social identity, and agents may be biased in the way they perceive others.

This model design allows us to investigate our main research question: does in-group bias impede or foster consensus among homophilic agents? It may seem obvious that in-group bias should create divisions between groups, inhibiting consensus formation. However, in-group bias may also speed up the convergence of opinions within in-groups, thus allowing opinions to spread further across the network before polarization can take hold [50,51]. This suggests that the structure of social interactions may moderate the effects of in-group bias. We thus ask: is the effect of in-group bias consistent over different network topologies? If not, what is the mechanism by which in-group bias can foster consensus? By setting up the model with different homophilic network topologies, we show that the in-group bias impedes consensus in societies if the topology is highly random. By contrast, the in-group bias fosters consensus in societies if the topology is highly clustered. These contrasting effects are robust outcomes unless homophily is very high or agents are strongly predisposed in their initial opinions such that an enhanced disagreement between the identity groups at the outset of the simulations inhibits the formation of consensus. The results of our model suggest that the impact of social identity and in-group bias can only be evaluated in relation to the underlying topology of the communication network.

## 2. Model description

### 2.1. Summary

**Figure 1.**
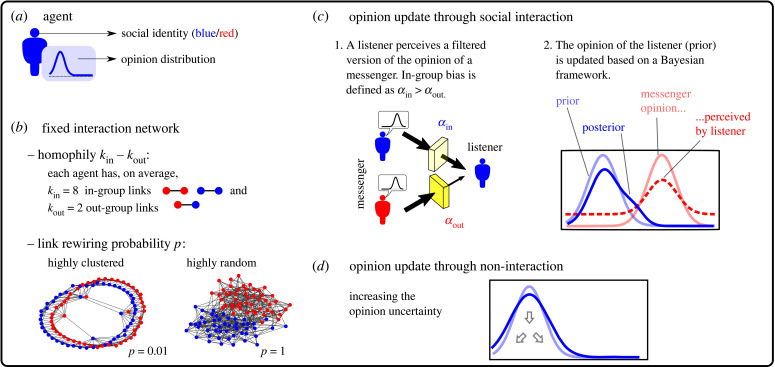
Schematic of (*a*) an agent with associated properties, (*b*) the interaction network, which remains unchanged throughout the simulation and is characterized by homophily and link rewiring (creating network topologies varying from highly clustered to highly random), and the opinion update process, which includes (*c*) social interaction or (*d*) non-interaction.

Our model simulates opinion formation in a small, virtual society consisting of *n* agents. Figure 1 shows a conceptual representation of the model and the effects of social identity on the model processes. Each agent represents an individual human being and is characterized by (i) an opinion on a disputed issue like climate change, expressed as a distribution and (ii) a social identity, expressed as an exclusive affiliation to one of two distinct groups (figure 1*a*). Agents are embedded in a fixed interaction network, which is characterized by (i) a degree of identity-driven homophily and (ii) a probability of link rewiring (figure 1*b*). The network topologies range from highly clustered to highly random. Over time, the opinions of all agents evolve via one of two processes. With a certain probability, a focal agent interacts with a linked neighbor and its opinion distribution is modified by that interaction. In-group bias involves that agents are more influenced by interactions with in-group members than by interactions with out-group agents (figure 1*c*). If the agent does not interact, its opinion distribution broadens, thus, increasing the susceptibility of that agent’s opinion to future social influences (figure 1*d*). In the following sections, we provide detailed explanations of these model features. With the objective of fostering reproducibility, transparency and flow of ideas, we make the model available as open-source software [52] so that it can be used, modified and redistributed freely.

### 2.2. Agent characteristics

Each agent is characterized by an opinion about the debated issue. With ‘opinion’, we mean the agent’s subjective point of view on the topic, such as its degree of concern about climate change. Opinions and beliefs are complex cognitive constructs, involving mental representation and rational processing of both direct evidence and social influence. For simplicity, we use the word ‘opinion’ in a way consistent with much of the opinion dynamics literature, in which opinions are updated through social influence and not direct experience. As such, our results will apply most readily to opinions shaped largely through social influence. Adopting a framework used in previous models [41,47,49], we represent the opinion of agent *i* at time *t* as a distribution *x*_*i*_(*b*, *t*) (*x*_*i*_ in the following) over the belief space B=[−1,1]. The opinions are initially assumed as Gaussian distributions characterized by a mean and a variance, the latter reflecting the agent’s uncertainty around the mean opinion. Over time, the shape of these distributions can change and may even become multi-modal. Each value *b* in the belief space represents a statement, for example, about climate change, ranging from *b* = −1 (‘I am not at all concerned about climate change’) to *b* = +1 (‘I am extremely concerned about climate change’). The opinion *x*_*i*_(*b*, *t*) represents the level of support of agent *i* for these statements *b* at time *t*. For computational reasons, we approximate the distribution and the belief space with 200 discrete, equally spaced values B={−0.995,−0.985,…,0.995}.

Each agent is also characterized by a social identity. This is expressed as an affiliation to one of two groups, red or blue, which can represent ‘youngsters’ and ‘elders’, for example. The social identity groups contain an equal number of agents. Social identities are visible to others and remain fixed throughout a simulation. Although in reality opinions often correlate with identities [53], we assume that the agents’ opinions are independent of their social identities (but relax this assumption later in §3.6 when we analyse a scenario in which social identities predispose the agents to specific opinions). The social identity of an agent defines its in-group—those agents sharing the same social identity—and the out-group—those with different social identities.

### 2.3. Interaction network

Agents are situated in an interaction network consisting of nodes and undirected, unweighted links. Nodes represent agents, and only agents connected through direct links can communicate. This reflects the fact that, in reality, most people have relatively few salient social influences that shape their opinions [13]. The network consists of two types of links: in-group links, connecting agents who share the same social identity, or out-group links, connecting agents with different social identities. In- and out-group links represent the same influence channels, although, in reality, these channels may have different characteristics. Assuming that social relationships evolve at a much slower pace than opinions, the network is created before the simulation begins and remains unchanged throughout the simulation. The network is defined by three parameters: (i) the average number of in-group links per agent, denoted as *k*_in_, (ii) the average number of out-group links per agent, denoted as *k*_out_, and (iii) the link rewiring probability, denoted as *p* (more on this parameter later). We define a network as homophilic if *k*_in_ > *k*_out_ (see [38,54] for similar conceptualizations of homophily). For instance, in a fully homophilic network with an average node degree of 10, the agents would have *k*_in_ = 10 in-group links and *k*_out_ = 0 out-group links, indicating a complete separation of the social identity groups. In a non-homophilic network, the agents would have, on average, *k*_in_ = 5 in-group links and *k*_out_ = 5 out-group links.

The network is constructed by creating two separate ring lattices, one for each social identity group. To establish in-group links, we connect each agent to its *k*_in_ nearest neighbors within its own group in the corresponding ring lattice.^1^ To establish out-group links, we connect each agent in the red group lattice to its *k*_out_ closest agents in the blue group lattice. For example, in a network with *n* = 100 agents, where agents 1–50 are red and agents 51–100 are blue, for *k*_out_ = 2, agent 1 is connected to its two closest out-group agents, 51 and 52, agent 2 is connected to agents 52 and 53, and so on (see electronic supplementary material, figure S1). After establishing this deterministic, homophilic network structure, all links are rewired with probability *p*. For the rewiring of in-group links, we follow the procedure described in the Watts–Strogatz model [55]. For the rewiring of out-group links, an existing link between agents *i* and *j* is substituted with a link between agent *i* and a randomly selected agent *k* from the out-group of agent *i*. This rewiring process preserves the total number of in-group and out-group links, thereby maintaining the degree of homophily in the network.

Homophily (*k*_in_ and *k*_out_) and rewiring (*p*) both play an important role in the topology of the network. In our main analysis, we focus primarily on networks with moderate homophily, specifically *k*_in_ = 8 and *k*_out_ = 2 (although we vary the degree of homophily in the sensitivity analysis). With this degree of homophily, we define a network as *highly clustered* when it is created with minimal or no rewiring (*p* → 0) and as *highly random* when it is created with maximum rewiring (*p* → 1). Highly clustered networks are characterized by a high clustering coefficient and a long average path length (see electronic supplementary material, figure S2). Moreover, they possess a regular structure (i.e. all agents have the same number of in- and out-group links) and a local topology (i.e. most of the neighbors of an agent are connected among themselves). Highly random networks are characterized by a low clustering coefficient and a short average path length, and the node degrees of the agents are heterogeneous (i.e. while some agents may be connected exclusively to in-group members, others may have many out-group links). Highly random networks resemble those generated by the stochastic block model [56] with a prescribed number of in- and out-group links.

### 2.4. Opinion update

At every time step, all *n* agents are selected asynchronously and in random order. With probability *q*, a selected agent (the listener) interacts with one of its neighbors (the messenger). With probability 1 − *q*, the listener does not interact, and its opinion distribution becomes more uncertain.

#### 2.4.1. Opinion update through social interaction

Interaction implies that the opinion of listener *i* changes based on observing the opinion of messenger *j*. We assume that the listener *i* only sees a distorted version of the messenger’s opinion distribution *x*_*j*_. Specifically, following a previous opinion formation model [49], the listener sees the distribution *x*_*j*_ with an uncertainty that is higher than the actual value. This reflects the fact that humans tend to evaluate others in a conservative way [57], perceiving their opinions as less conclusive than what they are. As described in the Introduction, social identity can undermine opinion change. For example, individuals may trust in-group members more, feel a stronger need to conform with them, or simply relate more to their in-group peers due to a shared language. To implement this bias, we assume that the degree of distortion depends on the social identity of the messenger. That is, for interactions with an in-group messenger *j*, listener *i* sees *x*_*j*_ as

2.1 $$p_{i}(x_{j})=\alpha_{\mathrm{in}}\cdot x_{j}+(1-\alpha_{\mathrm{in}})\cdot U,$$

with in-group perception *α*_in_ and, equivalently, with out-group perception *α*_out_ for interactions with out-group messengers. U denotes the uniform distribution on the belief space B. This perception step acts as a filter for *x*_*j*_ (figure 1*c*) and *α*_in/out_ represent the transparencies of the filters for in-/out-group messengers, respectively. A value of *α*_in/out_ = 1 (fully transparent filter) implies that the listener perceives *x*_*j*_ accurately, whereas *α*_in/out_ = 0 (fully opaque filter) implies that the listener perceives a uniform distribution that is unrelated to the messenger’s opinion. Negative values of *α*_in/out_ represent repulsive social influence, i.e. the listener would perceive the support of the messenger for a certain belief as counter-evidence for that belief. In this study, we restrict ourselves to positive values of *α*_in/out_.

We define agents as affected by in-group bias when their in-group perception is higher than their out-group perception (i.e. *α*_in_ > *α*_out_), meaning that a biased listener perceives the opinion of an in-group messenger as more certain than the same opinion of an out-group messenger. The in-group bias reflects a preference for accepting the opinions of in-group individuals over those of out-group individuals, with stronger bias indicating a larger disparity between the two. For simplicity, we assume that *α*_in_ and *α*_out_ are the same for all agents.

The opinion of the listener is updated following Bayesian calculus. In line with previous models [30,41,49,58], we consider the opinion distribution of the listener as the prior and the distorted version of the messenger’s opinion, as seen by the listener, as the likelihood. After an interaction with an in-group messenger *j*, the updated opinion of listener *i* becomes the Bayesian posterior (before normalization),

2.2 $$x_{i}\leftarrow x_{i}\cdot p_{i}(x_{j})=\alpha_{\mathrm{in}}\cdot x_{i}\cdot x_{j}+(1-\alpha_{\mathrm{in}})\cdot x_{i}\cdot U,$$

and, equivalently, with *α*_out_ after an interaction with an out-group messenger. Equation (2.2) encompasses two competing forces: (i) an assimilative force that pulls the opinion of the listener towards the opinion of the messenger and (ii) a conservative force that keeps the opinion of the listener unchanged, especially if the opinion of the messenger is very distant. The relative strengths of these forces depend on *α*_in_ and *α*_out_ and on the overlap between *x*_*i*_ and *x*_*j*_. Figure S3 in the electronic supplementary material provides an illustrative example of how these forces shape the posterior opinions of agents depending on *α*_in__/out_ and different messenger opinions.

#### 2.4.2. Opinion update through non-interaction

At each time step, only a fraction of the agents (on average *q* · *n*) change their opinions following social interactions. For the remaining agents, non-interaction slightly increases the uncertainty characterizing their opinion distributions. This reflects the fading of strong emotions or imperfect memory of arguments (see [37,41,59] for models with similar processes). The posterior opinion of a non-interacting agent is obtained by solving the diffusion heat equation for one time step *t* → *t* + 1 (using implicit differentiation via the backward time-centred space method),

2.3 $$\begin{array}{rl} \frac{d}{dt}\;x_{i}(b,t) & =\kappa \cdot \frac{d^{2}}{db^{2}}\;x_{i}(b,t)\\ \overset{\mathrm{discrete}}{\underset{\mathrm{solution}}{---⟶}}\;\;\frac{{x_{i}}_{b}^{t+1}-{x_{i}}_{b}^{t}}{\Delta t} & =\kappa \cdot \frac{{x_{i}}_{b+\Delta b}^{t+1}-2\cdot {x_{i}}_{b}^{t+1}+{x_{i}\;}_{b-\Delta b}^{t+1}}{\Delta b^{2}}, \end{array}$$

with zero Dirichlet boundary conditions at the edges of the belief space. This process depends on the parameter *κ*, which determines the speed with which the opinion distribution decays during non-interaction. While social interaction tends to narrow down opinion distributions, non-interaction broadens opinion distributions, making them more susceptible to change during future interactions. Consequently, if an agent does not interact for a sufficiently long time, it eventually adopts a uniform opinion distribution, indicating complete neutrality or indifference towards the issue. The impact of a more recent social interaction on an opinion, thus, tends to outweigh that of previous interactions.

### 2.5. Initialization and analysis of results

At the beginning of a simulation, *t* = 0, we create a society of *n* = 100 agents as follows. We divide the agents into two evenly sized social identity groups, red and blue, and situate them in a fixed network with a moderate level of homophily such that agents are on average linked to *k*_in_ = 8 in-group and *k*_out_ = 2 out-group members and with a specific degree of clustering determined by the rewiring probability *p*. Then, we initialize the agents with opinions represented as Gaussian distributions, $x_{i}∼N(\mu_{i,0},\sigma_{i,0})$, with fixed variance *σ*_*i*,0_ = *σ*_0_ = 0.2 and randomly sampled mean *μ*_*i*,0_ ∈ [−1, 1]. At the bounds of the belief space, the distributions are truncated and, therefore, mean and variance are not equivalent to those of the initial Gaussian distribution. This choice of opinion initialization allows us to distribute agent opinions uniformly over the belief space (except at the bounds), such that the initial state represents a very diverse and heterogeneous society. In §3.6, we explore an alternative scenario in which agents have predisposed initial opinions depending on their social identities, i.e. an agent *i* is initialized with a negative *μ*_*i*,0_ with a higher probability of 0.5 + *δ*/2 (with predisposition *δ*) if it has the red identity, and a lower probability of 0.5 − *δ*/2 if the agent has the blue identity. Finally, we fix the interaction probability *q* = 0.2 and the opinion decay speed during non-interaction *κ* = 0.0002. We analyse the robustness of our assumptions by varying the values of these parameters in §§3.4–3.6.

Our main analysis focuses on comparing the opinion patterns obtained with different in-group and out-group perceptions and, thus, also different strengths of in-group bias. To avoid potential divisions by 0 when normalizing the posterior in equation (2.2), we choose *α*_in__/out_ ∈ [0, 0.99]. For illustrative purposes, we later focus on a set of distinct values of *α*_in_ and *α*_out_. We consider three particular cases, *U*_1_, *U*_2_ and *U*_3_, representing societies of unbiased agents, and one case, *B*, representing a society of biased agents. There are three unbiased cases because agents can be characterized by different levels of perception. Regardless of social identity, agents in *U*_1_ are ‘skeptical’ towards others (*α*_in_ = *α*_out_ = 0.25), agents in *U*_2_ are ‘neutral’ towards others (*α*_in_ = *α*_out_ = 0.5), and agents in *U*_3_ are ‘credulous’ towards others (*α*_in_ = *α*_out_ = 0.75). By contrast, agents in societies *B* are ‘credulous’ towards in-group agents (*α*_in_ = 0.75) and ‘skeptical’ towards out-group agents (*α*_out_ = 0.25). Comparing the results of *U*_1_, *U*_2_ and *U*_3_ with those of *B* allows us to isolate the effects of the bias.

We start our analysis by describing the specific opinion patterns produced by the model in general terms (§3.1). Then, we systematically analyse how often societies reach a consensus after a fixed number of *t* time steps, i.e. the consensus frequency, *C*_*t*_, for societies characterized by highly random (§3.2) or highly clustered networks (§3.3). There are many ways to quantify disagreement or consensus [60–63]. Here, we measure the level of disagreement with the standard deviation, *σ*, of the opinion means of all agents and we define consensus when *σ* is below a threshold *σ*_cons_ = 0.01. Simulations are terminated after *t* = 5000 steps, when an agent has, on average, updated its opinion 1000 times during one-on-one interactions (with interaction probability *q* = 0.2). The results do not change qualitatively for shorter and longer simulation times (see §3.4). Because the model is stochastic with respect to network topology (if *p* > 0), update order, and initial opinions, we present consensus frequencies, *C*_t_, as averages over ensemble runs of 1000 realizations of a society with the same parameter configuration but different random seeds.

## 3. Results

### 3.1. Consensus can emerge through an abrupt, stochastic transition

**Figure 2.**
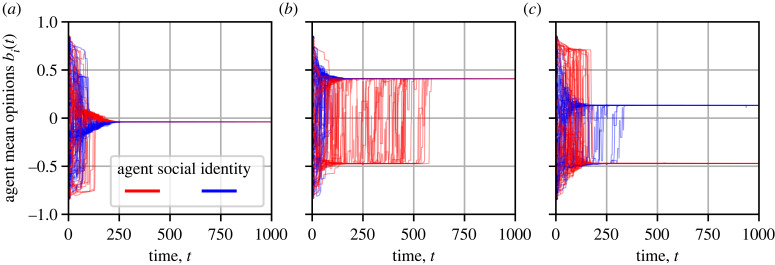
Time evolution of the agents’ mean opinions in three example simulations of a society *B* composed of biased agents with in-group perception *α*_in_ = 0.75 and out-group perception *α*_out_ = 0.25, and an interaction network characterized by moderate homophily, *k*_in_ = 8 and *k*_out_ = 2, and maximum link rewiring, *p* = 1. Panels *a*–*c* show realizations of the same society but with different random seeds, which affects (i) the initial conditions, (ii) the network topology, and (iii) the update order. In these examples, opinions (*a*) converge quickly, at *t* = 198, to a consensus, (*b*) separate into two distinct clusters, corresponding to the two social identity groups, that converge to a consensus at *t* = 585, when one opinion cluster absorbs the other, or (*c*) separate into two distinct clusters that remain separate over the entire simulation.

Figure 2 shows the temporal evolution of opinions in three example simulations of a society *B* composed of biased agents with *α*_in_ = 0.75 and *α*_out_ = 0.25. The societies in these examples are characterized by homophilic and highly random networks, i.e. *k*_in_ = 8, *k*_out_ = 2 and *p* = 1. In the early phase of a simulation, opinions converge within small local neighborhoods. This leads to the formation of opinion clusters. Neighborhoods in the highly random network are densely interconnected such that these clusters typically dissolve quickly, causing fast overall convergence towards a relatively moderate consensus opinion (figure 2*a*). If, however, distinct clusters become sufficiently large and the convergence within these clusters dominates over the alignment among different clusters, then disagreement stabilizes and fast consensus is prevented (figure 2*b*,*c*). Disagreement, however, is a transient, meta-stable state of the system. In some cases (e.g. figure 2*b*), alignment pressures among the opinion clusters dominate over their internal cohesion. This leads to an abrupt, stochastic transition towards a consensus where one cluster absorbs the other and the resulting consensus opinion tends to be more extreme than when consensus is reached very early in the simulation (as in figure 2*a*; see electronic supplementary material, figure S4). In other cases (e.g. figure 2*c*), disagreement persists because opinion clusters turn into echo chambers, in which the agents are confronted almost exclusively with similar opinions. Over time the agents become increasingly certain about their opinions (i.e. the variances of their opinion distributions narrows down) and, thus, unresponsive to agents with different opinions. In this scenario, we find that the standard deviation of all mean opinions, *σ*, is typically in the range from 0.2 to 0.6, similar to its initial value at *t* = 0 (and thus disagreement and consensus are qualitatively distinct patterns regardless of the exact value of the threshold, *σ*_cons_ = 0.01).

### 3.2. In-group bias impedes consensus in homophilic, highly random networks

**Figure 3.**
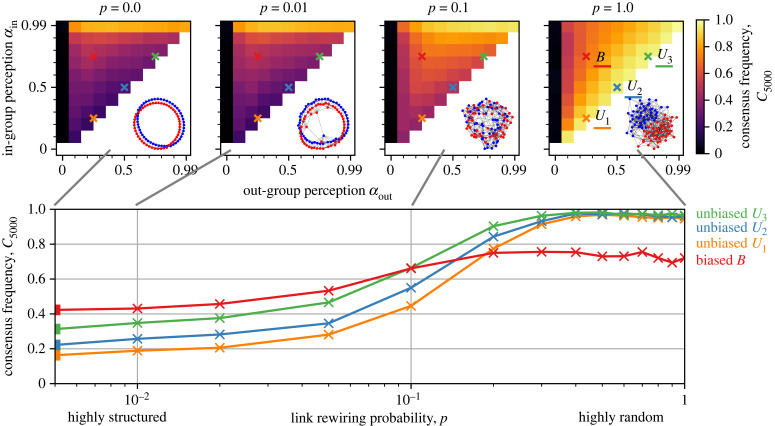
Consensus frequency *C*_5000_ before *t* = 5000 time steps for societies characterized by networks with homophily, *k*_in_ = 8 and *k*_out_ = 2, and different link rewiring probabilities *p* ∈ [0, 1]. In highly random networks (*p* ≫ 0.1), consensus occurs less often in a society *B* of biased agents (red) than in societies *U*_1_ (orange), *U*_2_ (blue) or *U*_3_ (green) of unbiased agents. However, in highly clustered networks (*p* < 0.1), a society *B* reaches consensus more frequently than societies *U*_1_, *U*_2_ or *U*_3_. The inset panels *p* = 0, *p* = 0.01, *p* = 0.1, and *p* = 1, show the consensus frequencies over the full parameter space of *α*_in_ and *α*_out_, where societies of unbiased agents, such as *U*_1_, *U*_2_ and *U*_3_, are located on the diagonal and societies of biased agents, such as *B*, are located in the upper left corner. With increasing bias (i.e. moving further away from the diagonal), consensus frequencies consistently decrease or increase for, respectively, highly random or highly clustered networks, unless *α*_out_ → 0. Each inset panel also shows an example of a corresponding network topology, with the node colour (blue and red) representing the two social identities.

Figure 3 shows the frequency, *C*_5000_, with which societies in our ensemble simulations reach a consensus within *t* = 5000 steps, depending on the link rewiring probability, *p*, in the network and the agents’ in- and out-group perception, *α*_in_ and *α*_out_ (in particular, for societies *U*_1_, *U*_2_, *U*_3_ and *B*). We first focus on societies characterized by highly random networks, *p* ≫ 0.1. In general, consensus is frequently reached within *t* = 5000 time steps unless the out-group perception of the agents is very low, *α*_out_ → 0. However, consensus occurs somewhat less frequently if the agents are biased (upper left part of panel *p* = 1 in figure 3) than if they are unbiased (diagonal part of panel *p* = 1), regardless of the exact values for *α*_in_ and *α*_out_. For example, for *p* = 1, societies *U*_1_, *U*_2_ and *U*_3_ reach consensus within 5000 time steps in, respectively, 95%, 96% and 97% of the simulations, but society *B* reaches consensus ‘only’ in 72% of the simulations. These numbers increase only marginally over much longer simulation times (see §3.4). Biased agents see opinions of in-group messengers as more certain and, in homophilic networks, they also tend to interact more with in-group members. This combination facilitates and stabilizes the emergence of echo chambers, which mostly coincide with the identity groups (figure 2*c*). In sum, in-group bias impedes consensus in homophilic and highly random networks.

### 3.3. In-group bias fosters consensus in homophilic, highly clustered networks

In contrast to impeding consensus in societies with highly random networks (*p* ≫ 0.1), in-group bias promotes consensus in societies with highly clustered networks (*p* < 0.1, figure 3). In general, higher clustering in the network, i.e. fewer rewired links, reduces the consensus frequency regardless of *α*_in_ and *α*_out_. The reason for this is that paths between agents in a clustered network are typically longer than in a random network, which leads to weaker influence between them and to longer convergence times. However, unbiased agents are much more affected by clustering than biased agents. For example, in highly clustered networks with *p* = 0, a consensus is reached in 42% of a society *B* (72% for *p* = 1), but only in 22% of a society *U*_2_ (96% for *p* = 1). This qualitative pattern remains robust over all time scales, even if societies characterized by such clustered networks reach consensus at time scales beyond *t* = 5000 steps (see §3.4 for more details). Note that this positive effect of the in-group bias on consensus formation holds only for societies in which the agents are at least somewhat susceptible to out-group influences, *α*_out_ > 0 (figure 3).

**Figure 4.**
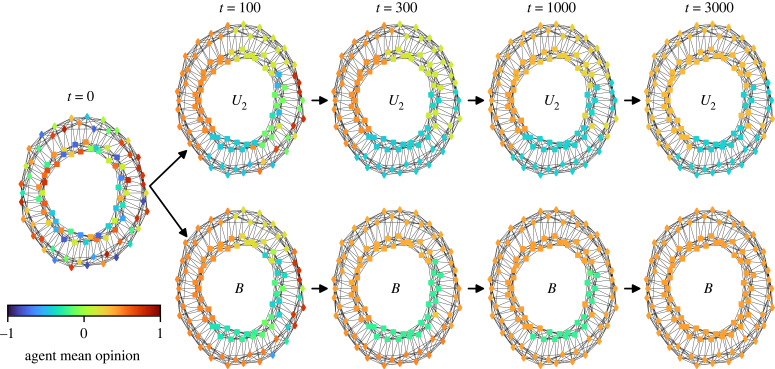
Example simulation for a society *U*_2_ of unbiased, ‘neutral’ agents (*α*_in_ = *α*_out_ = 0.5) and a society *B* of biased agents (*α*_in_ = 0.75 and *α*_out_ = 0.25) where the society is characterized by a homophilic and highly clustered interaction network, *k*_in_ = 8 and *k*_out_ = 2, and *p* = 0. The shapes and positions of the nodes (outer and inner circle) represent the agents’ social identities. The colours of the symbols represent the mean opinions of the agents. In *U*_2_ (upper panels), a small, isolated opinion cluster emerges (light blue) and turns into a minority echo chamber that remains stable beyond *t* = 3000. In *B* (lower panels), the relatively higher alignment pressure among in-group members prevents the formation of isolated opinion clusters. One social identity group (the outer circle) reaches a consensus, which the out-group agents align to before *t* = 3000. Consensus is thus reached in society *B*, but not in society *U*_2_. In this particular example, consensus is also not reached in the other societies of unbiased agents, *U*_1_ and *U*_3_ (see electronic supplementary material, figure S5).

This result, that in-group bias can promote consensus in highly clustered networks (*p* < 0.1), may appear counter-intuitive. For a better understanding, we provide a video in the electronic supplementary material that shows a typical simulation for the societies *U*_1_, *U*_2_, *U*_3_ and *B*, all characterized by a homophilic and highly clustered network (*p* = 0) and an identical configuration of initial opinions. Figure 4 shows snapshots of the society *B*, which reaches consensus, and of the society *U*_2_, which does not reach consensus. In *U*_2_, isolated opinion clusters can form across social identity divisions, with the effect that the agents are fully detached from contrasting opinions elsewhere in the network. Therefore, opinions are clustered by space rather than social identity in this scenario. In *B*, the alignment pressure exerted by the in-group outweighs the alignment pressure exerted by neighboring out-group agents, even when the path length between the in-group agents tends to be large. Agents are, thus, more likely to reach a consensus within their in-group and the possibility for the formation of isolated echo chambers is reduced. Once one social identity group has reached a consensus, the agents from the other group are collectively pulled to that consensus opinion, and, eventually, a society-wide consensus is established. This pattern of opinion dynamics is fostered by the in-group bias affecting agents in societies *B* but not agents in societies *U*_1,2,3_.

### 3.4. The interplay between in-group bias and network clustering is a robust pattern

**Figure 5.**
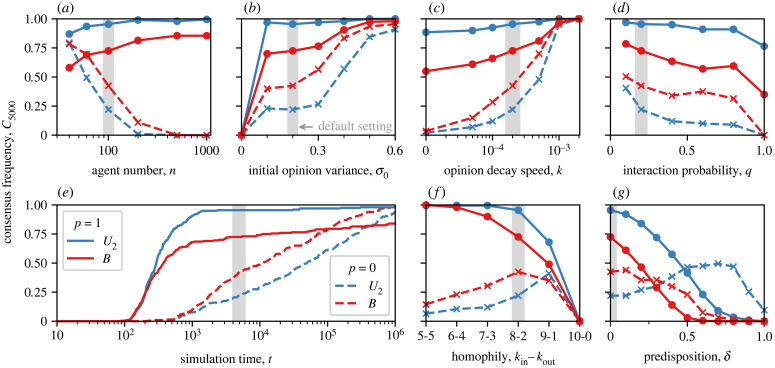
Sensitivity analysis for the consensus frequency *C*_5000_ of societies *U*_2_ (blue lines) and *B* (red lines) characterized by highly random networks (*p* = 1, solid lines with dots) or highly clustered networks (*p* = 0, dashed lines with crosses). The parameters investigated are (*a*) the number of agents, *n*, (*b*) the variance of the initial Gaussian opinion distributions, *σ*_0_, (*c*) the opinion decay speed, *κ*, during non-interaction (*d*) the probability of interaction, *q*, (*e*) the simulation time, *t*, (*f*) the degree of homophily, *k*_in_ and *k*_out_, and (*g*) the predisposition of the initial opinions, *δ*. The vertical grey areas mark standard parameter values used for obtaining the results presented in the previous figures (*n* = 100, *σ*_0_ = 0.2, *κ* = 0.0002, *q* = 0.2, *t* = 5000, *k*_in_ = 8, *k*_out_ = 2 and *δ* = 0). While the parameter choices affect the exact values of the consensus frequencies, the general results are robust. Specifically, the in-group bias affecting the agents in societies *B* prevents consensus in highly random networks (the red solid lines are always below the blue solid lines) and the bias fosters consensus in highly clustered networks (the red dashed lines are always above the blue dashed lines). The latter holds true in all but two extreme cases, where societies are characterized by very strong homophily, *k*_in_ ≥ 9 and *k*_out_ ≤ 1, or high predisposition *δ* ≥ 0.4.

Our main results—in-group bias impedes consensus in highly random networks (§3.2) and fosters consensus in highly clustered networks (§3.3)—are robust under a wide range of parameter values. Figure 5 shows the consensus frequencies for highly random networks (*p* = 1, dots), and for highly clustered networks (*p* = 0, crosses) at different values of all model parameters. For *p* = 1, the consensus frequencies obtained in societies *B* of biased agents (red solid lines in figure 5), are reliably smaller than those in societies *U*_2_ of unbiased, ‘neutral’ agents (blue solid lines). Similarly, for *p* = 0, the consensus frequencies in societies *B* (red dashed line in figure 5), are reliably larger than those in societies *U*_2_ (blue dashed lines) with only two exceptions for extreme homophily or for strong predisposition (more details in §§3.5 and 3.6), or for both combined (see electronic supplementary material, figure S6).

While different values of *n*, *σ*_0_, *κ*, *q* and *t* do not change the general results of the study, some interesting effects are discernible at a higher level of detail. First, a larger number of agents, *n*, increases or decreases the consensus frequency depending on the network topology (figure 5*a*). Very large societies virtually always reach consensus when the interaction network is highly random (*p* = 1), but they barely reach consensus within *t* = 5000 time steps when the network is highly clustered (*p* = 0). Second, the parameters *σ*_0_, *κ* and *q* (figure 5*b*–*d*) determine how the uncertainties of the opinion distributions evolve and are, thus, crucial for consensus formation. The formation of consensus is facilitated by high uncertainties in the Gaussian initial opinions, fast decay of opinion distributions during non-interaction and reduced frequency of interactions. Finally, the consensus frequencies depend on the simulation time scale (figure 5*e*). Consensus may emerge already after *t* = 100 time steps. In highly random networks, consensus frequencies reach high values relatively quickly, but in highly clustered networks consensus forms much slower and may still emerge after a much longer simulation time. Eventually, for the agents in society *B*, consensus becomes virtually certain in highly clustered networks (*p* = 0, dashed) but the consensus frequency saturates at $C_{10^{6}}=84\;\%$ in highly random networks (solid red line)

### 3.5. Moderate homophily promotes consensus in highly clustered networks

The presented results are based on interaction networks with moderate homophily, specifically, where agents have, on average, *k*_in_ = 8 in-group links and *k*_out_ = 2 out-group links. The main results (that the in-group bias has opposite effects on consensus formation depending on the random or clustered network topology, see §§3.2 and 3.3) also hold true for less homophilic or even non-homophilic networks (figure 5*f*). Only in networks characterized by very strong homophily, *k*_in_ > 9 and *k*_out_ < 1 (with an average of 10 links per agent), does in-group bias impede consensus in both random and clustered network topologies. Homophily by itself has diverse effects on consensus formation, regardless of the in-group bias. Increasing homophily impedes consensus in highly random networks (*p* = 1, solid lines in figure 5*f*), but it promotes consensus in highly clustered networks (*p* = 0, dashed lines) unless the two groups are (nearly) fully separated.

### 3.6. Predisposition of unbiased agents promotes consensus in homophilic, highly clustered networks

The presented results are based on the assumption that opinions are uniformly distributed at the start of a simulation and independent of the agents’ social identities. We, therefore, tested the case of predisposed agents (figure 5*g*), in which the initial opinions of agents with different social identities tend to be positioned on opposite sides of the belief space (see electronic supplementary material, figure S7, for a more detailed description). Our main results hold true for weak predisposition, but not for strong predisposition. In the latter case, in-group bias impedes consensus in both random and clustered networks. In general, predisposition impedes consensus. Surprisingly, however, predisposition (up to *δ* = 0.7) can promote consensus in highly clustered networks, but only when the agents are unbiased (society *U*_2_, dashed blue line).

## 4. Discussion

Mathematical modelling serves various purposes in the social sciences [64–66]. While theories of human behaviour remain largely verbal and ambiguous, idealized modelling can be an important step in the development of more detailed theories and towards a better understanding of the social phenomena (see [67–69] for further discussions). For example, a large and influential literature on opinion dynamics uses abstract, idealized models of social influence to explore factors that lead to phenomena such as consensus, polarization, fractionalization and extremism. Our modelling study falls into this tradition, yet our contribution is to point out how in-group bias—a common and well-documented facet of human behaviour—influences opinion dynamics in important and non-obvious ways. We have thus presented an agent-based model of opinion formation based on social influence with the social identities of the agents driving their interactions and perceptions. In line with theoretical arguments about the importance of social identity [7,15,29,70,71] and with empirical studies indicating that identity may be a crucial driver of polarization [20,72–74], opinion patterns in our model are crucially shaped by social identity. The model shows how in-group bias can have different societal-level effects depending on the network structure. Specifically, in-group bias prevents consensus in highly random networks, but fosters consensus in highly clustered networks—as long as homophily and predisposition are not extreme.

The outcome of consensus or disagreement in the model depends on the interplay between two forces during social interactions: an assimilative force, acting to align opinions and narrow them down, and a conservative force, acting to preserve the original opinions. These two forces and their interplay are common in many opinion dynamics models (e.g. [40,62,75,76]), but in contrast to most of these studies, we do not assume that agents fully ignore others (as e.g. in bounded confidence models [77,78]) or that opinions diverge when their disagreement exceeds some threshold (as e.g. in [60,79]). In our model, agents biased with respect to social identity adapt more promptly to in-group members than to out-group members because they perceive in-group opinions as more conclusive than out-group opinions. This purely positive social influence implies that consensus is an inevitable outcome (as in other studies, such as [30,40,80]; with purely assimilative influences). However, to reach a consensus in a reasonable time, the agents need to remain sufficiently susceptible to opinion change. Disagreement can solidify and persist in our model only when agents are trapped in isolated opinion clusters and when those clusters turn into echo chambers, inside which the agents’ opinions become increasingly narrow. This outcome is in line with empirical evidence that echo chambers play an important role in political debates [81,82] including the debate on climate change [83,84]. Moreover, disagreement can be a robust outcome of our model without opinion polarization. This pattern is consistent with survey-based research [20,72] showing that, for example, US citizens are divided more along social identity lines than actual opinion differences.

Our model shows that, in the long run, in-group bias prevents consensus in societies characterized by networks with low clustering, i.e. with highly random link structures. In such networks, the average path length, especially between in-group members, is short, thus fostering in-group alignment. The bias exacerbates this effect by turning opinion clusters more easily into echo chambers and, thereby, consolidating disagreement between identity groups. This view is consistent with the notion that in-group bias is negatively associated with consensus building (e.g. [72,85,86]). A similar effect of an identity-related bias was described in a previous modelling study [60] with agents characterized by different demographic attributes that influenced whether they aligned to or repelled from the opinions of others. Similar to Flache & Mäs [60], we find that a strong in-group bias generates disagreement but, in contrast to them, we obtain this result without including repulsive social influences between dissimilar agents.

The most important result of our study is that in-group bias can have opposite effects depending on the topology of the communication network. High clustering in the network inhibits consensus formation in general. However, in contrast to the result discussed above, the presence of in-group bias facilitates consensus in this case. In such highly clustered networks, the average path length between agents tends to be long, such that agents at opposite ends of the network exert little influence on each other. Consequently, local opinion clusters emerge, irrespective of social identities, which inhibit a society-wide consensus. In this case, opinions are separated by space, and in-group bias helps to dissolve such clusters, thus, promoting consensus. This view is consistent with the notion that, under certain conditions, in-group bias is positively associated with consensus building (e.g. [87]) and with empirical evidence showing that concerns about climate change have increased faster among younger generations than older generations [88]. In our model, the mechanisms by which the in-group bias leads to consensus are analogous to those considered by Mäs *et al.* [37]. Mäs *et al.* [37] used the model of Flache & Mäs [60] with the addition of homophily in relation to the way agents choose their interacting partners. The presence of in-group bias and homophily caused the agents to moderate extremists within their respective in-groups, which then promoted consensus among these moderate groups in the long run. Our results exhibit the same two-step process in highly clustered networks, but we also find that besides homophily and bias, this process can be driven solely by the topology of the communication network. The importance of network topology in relation to identity was recently observed also in a two-armed bandit game model [89] in which agents learned from their peers but distrusted information from out-group members. Similar to our results, the (very simple) social networks considered by Fazelpour & Steel [89] determined whether the bias prevented or promoted collective performance.

Although the positive effect of the bias on consensus formation in highly clustered networks is robust over a wide range of parameter configurations, there are some limitations in two extreme cases: strong homophily and marked predisposition. In highly clustered networks, homophily and predisposition affect opinion formation similar to in-group bias. They promote the convergence of opinions among in-group members that are dispersed over the network, thereby, homophily and predisposition can foster consensus. Although counter-intuitive at first sight, this result reflects the well-established concept that any process that prevents social learners from converging too quickly towards a local optima can improve the collective performance [36,51,90]. However, the combination of in-group bias with either strong homophily or marked predisposition (or a combination of the two) reverses this positive effect and prevents consensus formation.

Social identities and related biases are particularly relevant in the debate on climate change [12,91]. As discussed before, climate change entails an intergenerational conflict [88,92,93], which is commonly addressed in the debate (‘We young people [ … ] must hold the older generations accountable for the mess they have created [ … ].’—Greta Thunberg^2^). There is ample empirical evidence on the fact that social identity affects the way people perceive the opinions of others on topics like climate change [28,73,94]. This evidence suggests that it may be easier to dismiss alarmed statements from an out-group contact as ‘merely their opinion’, given that such opinions are subjective rather than rooted in logic- or evidence-based reasoning. Recent studies have proposed strategies to reduce the relevance of social identities and related biases in political debates, for example, by exposing people to trusted expert opinions, like those presented in the IPCC report [20], by emphasizing non-political similarities between different groups, like connecting people with similar musical taste [6], or by fostering contact between members of different groups to reduce prejudices [95]. Analogously, other studies (e.g. [96]) have proposed strategies that instead aim to highlight social identity and exploit related biases in order to overcome disagreement in political debates. An example of such a strategy consists of channelling policy communication through in-group specific messengers, such as Greta Thunberg [97–99]. Another example is the use of large social identity groups to reinforce people’s perceived efficacy (by the sheer size of the group) during global crises [98,100].

Over the past years, the generational identity gap and the associated conflicts have arguably increased as youth movements surged in popularity across the globe [23,101]. Has this social identity contributed to a shared view of climate change among youngsters and beyond? Or have social identities and related in-group biases further polarized society? Answers to these questions remain elusive. Models, like the one we have presented here, are, by definition, approximations of reality. And although they cannot include the broad spectrum of processes and cognitive biases characterizing our society, their simplicity enables us to test mechanisms that can generate different macroscopic patterns such as consensus or disagreement [102]. Our model shows that in-group bias can have such opposing effects depending on the communication network characterizing a society. While it is relatively well established that networks of social influence are somewhat affected by homophily [103], more fine-grained aspects of the network topology are arguably less clear. For example, connections between users of social media platforms such as ‘Twitter’ (now ‘X’), are driven by identity, but the network comprises a mix of short- and long-range connections, and local associations remain important [104]. We show here that these aspects play a crucial role in the emergence of opinion patterns. Sparse long-range connections and strong local clustering generally impede consensus. Under such conditions, in-group bias can stimulate consensus formation. Contrastingly, the bias impedes consensus formation in networks with many long-range and less locally confined connections (similar, for example, to internet forums). The effects of in-group bias are thus moderated by network structure. This is important because real-world network structures are quite diverse, as the analysis of networks in social media platforms like ‘Twitter’ suggests [103,105], which can lead to unanticipated communication and opinion patterns.

Future research could focus on investigating more closely the network structures that are characteristic of certain real-world debates, and in particular their clustering and path length properties. This research may indicate which of the effects of biases related to social identity dominate in such debates. How heterogeneity in the degrees of in-group bias or differences in the agents’ social power in the network can alter the effects of in-group bias could be another interesting avenue for future research. Although intergroup relations are a key factor in driving opinion formation in society and, thus, for example, in facilitating or hampering the design of effective climate change mitigation policies [12,86,93], a surprisingly low number of modelling studies have tackled the problem. Ultimately, by shedding new light on the contrasting effects that the interaction among social identity, in-group bias and network topology can have on opinion dynamics, we hope that our study can inspire additional modelling work in this field.

## Data Availability

All model code is made available from the Zenodo repository: https://doi.org/10.5281/zenodo.10118407 [52]. We make the model available as open-source software so that it can be used, modified and redistributed freely. Further analysis is provided in electronic supplementary material [106].

## Endnotes

1 Note that if *k*_in_ is uneven, for example, *k*_in_ = 5, then we connect each agent to its two closest agents in both clockwise and counter-clockwise directions in the ring lattice and, with probability 1/2, additionally to the third closest agent in the clockwise direction, such that the average in-group link degree is *k*_in_ = 5.

2 CNN (@CNN) ‘We must hold the older generations accountable for the mess they have created … and say to them you cannot continue risking our future like this.' Teen climate activist Greta Thunberg calls on young people to use their anger as activism.

## References

[1] Nowak A, Szamrej J, Latané B. 1990 From private attitude to public opinion: a dynamic theory of social impact. Psychol. Rev. **97**, 362-376. (10.1037/0033-295X.97.3.362)

[2] Moussaïd M, Kämmer JE, Analytis PP, Neth H. 2013 Social influence and the collective dynamics of opinion formation. PLoS ONE **8**, e78433. (10.1371/journal.pone.0078433) 24223805 PMC3818331

[3] Barnes ML, Wang P, Cinner JE, Graham NAJ, Guerrero AM, Jasny L, Lau J, Sutcliffe SR, Zamborain-Mason J. 2020 Social determinants of adaptive and transformative responses to climate change. Nat. Clim. Change **10**, 823-828. (10.1038/s41558-020-0871-4)

[4] Dunlap RE, McCright AM, Yarosh JH. 2016 The political divide on climate change: partisan polarization widens in the U.S. Environ. Sci. Policy for Sustainable Dev. **58**, 4-23. (10.1080/00139157.2016.1208995)

[5] Milosh M, Painter M, Sonin K, Van Dijcke D, Wright AL. 2021 Unmasking partisanship: polarization undermines public response to collective risk. J. Public Econ. **204**, 104538. (10.1016/j.jpubeco.2021.104538)

[6] Balietti S, Getoor L, Goldstein DG, Watts DJ. 2021 Reducing opinion polarization: effects of exposure to similar people with differing political views. Proc. Natl Acad. Sci. USA **118**, e2112552118. (10.1073/pnas.2112552118) 34937747 PMC8719860

[7] Pearson AR, Schuldt JP, Romero-Canyas R. 2016 Social climate science: a new vista for psychological science. Perspect. Psychol. Sci. **11**, 632-650. (10.1177/1745691616639726) 27694459

[8] Kjeldahl EM, Hendricks VF. 2018 The sense of social influence: pluralistic ignorance in climate change. EMBO Rep. **19**, e47185. (10.15252/embr.201847185) 30348889 PMC6216292

[9] Bak-Coleman JB et al. 2021 Stewardship of global collective behaviour. Proc. Natl Acad. Sci. USA **118**, e2025764118. (10.1073/pnas.2025764118) 34155097 PMC8271675

[10] Wallis H, Loy LS. 2021 What drives pro-environmental activism of young people? A survey study on the Fridays For Future movement. J. Environ. Psychol. **74**, 101581. (10.1016/j.jenvp.2021.101581)

[11] Kendal RL, Boogert NJ, Rendell L, Laland KN, Webster M, Jones PL. 2018 Social learning strategies: bridge-building between fields. Trends Cogn. Sci. **22**, 651-665. (10.1016/j.tics.2018.04.003) 29759889

[12] Pearson AR, Schuldt JP. 2018 Climate change and intergroup relations: psychological insights, synergies, and future prospects. Group Process. Intergr. Relat. **21**, 373-388. (10.1177/1368430217747750)

[13] Bond RM, Fariss CJ, Jones JJ, Kramer ADI, Marlow C, Settle JE, Fowler JH. 2012 A 61-million-person experiment in social influence and political mobilization. Nature **489**, 295-298. (10.1038/nature11421) 22972300 PMC3834737

[14] Price V. 1989 Social identification and public opinion: effects of communicating group conflicts. Public Opin. Quart. **53**, 197-224. (10.1086/269503)

[15] Smaldino PE. 2022 Models of identity signaling. Curr. Direct. Psychol. Sci. **31**, 231-237. (10.1177/09637214221075609)

[16] Fielding KS, Hornsey MJ. 2016 A social identity analysis of climate change and environmental attitudes and behaviors: insights and opportunities. Front. Psychol. **7**, 121. (10.3389/fpsyg.2016.00121) 26903924 PMC4749709

[17] Estrada M, Schultz PW, Silva-Send N, Boudrias MA. 2017 The role of social influences on pro-environment behaviors in the San Diego region. J. Urban Health: Bull. New York Acad. Med. **94**, 170-179. (10.1007/s11524-017-0139-0) PMC5391335 28265806

[18] Tajfel H. 1974 Social identity and intergroup behaviour. Soc. Sci. Inform. **13**, 65-93. (10.1177/053901847401300204)

[19] Hornsey MJ. 2008 Social identity theory and self-categorization theory: a historical review. Soc. Personal. Psychol. Compass **2**, 204-222. (10.1111/j.1751-9004.2007.00066.x)

[20] Flores A et al. 2022 Politicians polarize and experts depolarize public support for COVID-19 management policies across countries. Proc. Natl Acad. Sci. USA **119**, e2117543119. (10.1073/pnas.2117543119) 35042779 PMC8784107

[21] Hall MP, Lewis NA, Ellsworth PC. 2018 Believing in climate change, but not behaving sustainably: evidence from a one-year longitudinal study. J. Environ. Psychol. **56**, 55-62. (10.1016/j.jenvp.2018.03.001)

[22] Hornsey MJ, Harris EA, Bain PG, Fielding KS. 2016 Meta-analyses of the determinants and outcomes of belief in climate change. Nat. Clim. Change **6**, 622-626. (10.1038/nclimate2943)

[23] Gonyea JG, Hudson RB. 2020 In an era of deepening partisan divide, what is the meaning of age or generational differences in political values?. Public Policy & Aging Report **30**, 52-55. (10.1093/ppar/praa003)

[24] Lazarsfeld PF, Merton RK. 1954 Friendship as a social process: a substantive and methodological analysis. In *Freedom and control in modern society* (eds M Berger, T Abel, CH Page), pp. 18–66. New York, NY: D. Van Nostrand Company.

[25] McPherson M, Smith-Lovin L, Cook JM. 2001 Birds of a feather: homophily in social networks. Annu. Rev. Sociol. **27**, 415-444. (10.1146/annurev.soc.27.1.415)

[26] Brewer MB. 1979 In-group bias in the minimal intergroup situation: a cognitive-motivational analysis. Psychol. Bull. **86**, 307-324. (10.1037/0033-2909.86.2.307)

[27] Turner JC, Wetherell MS, Hogg MA. 1989 Referent informational influence and group polarization. Brit. J. Soc. Psychol. **28**, 135-147. (10.1111/j.2044-8309.1989.tb00855.x)

[28] Mackie DM, Gastardo-Conaco MC, Skelly JJ. 1992 Knowledge of the advocated position and the processing of in-group and out-group persuasive messages. Pers. Soc. Psychol. Bull. **18**, 145-151. (10.1177/0146167292182005)

[29] Hewstone M, Rubin M, Willis H. 2002 Intergroup bias. Annu. Rev. Psychol. **53**, 575-604. (10.1146/annurev.psych.53.100901.135109) 11752497

[30] Bartels LM. 2002 Beyond the running tally: partisan bias in political perceptions. Polit. Behav. **24**, 117-150. (10.1023/A:1021226224601)

[31] Richerson P et al. 2016 Cultural group selection plays an essential role in explaining human cooperation: a sketch of the evidence. Behav. Brain Sci. **39**, e30. (10.1017/S0140525X1400106X) 25347943

[32] Powell M, Kim AD, Smaldino PE. 2023 Hashtags as signals of political identity: #BlackLivesMatter and #AllLivesMatter. PLoS ONE **18**, e0286524. (10.1371/journal.pone.0286524) 37289780 PMC10249887

[33] Moser D, Steiglechner P, Schlueter A. 2022 Facing global environmental change: the role of culturally embedded cognitive biases. Environ. Dev. **44**, 100735. (10.1016/j.envdev.2022.100735)

[34] Brewer MB. 1999 The psychology of prejudice: ingroup love or outgroup hate? J. Soc. Issues **55**, 429-444. (10.1111/0022-4537.00126)

[35] Smaldino PE, Janssen MA, Hillis V, Bednar J. 2017 Adoption as a social marker: innovation diffusion with outgroup aversion. J. Math. Sociol. **41**, 26-45. (10.1080/0022250X.2016.1250083)

[36] Galesic M et al. 2023 Beyond collective intelligence: collective adaptation. J. R. Soc. Interface **20**, 20220736. (10.1098/rsif.2022.0736) 36946092 PMC10031425

[37] Mäs M, Flache A, Takács K, Jehn KA. 2013 In the short term we divide, in the long term we unite: demographic crisscrossing and the effects of faultlines on subgroup polarization. Organ. Sci. **24**, 716-736. (10.1287/orsc.1120.0767)

[38] Dandekar P, Goel A, Lee DT. 2013 Biased assimilation, homophily, and the dynamics of polarization. Proc. Natl Acad. Sci. USA **110**, 5791-5796. (10.1073/pnas.1217220110) 23536293 PMC3625335

[39] Smaldino PE, Jones JH. 2021 Coupled dynamics of behaviour and disease contagion among antagonistic groups. Evol. Human Sci. **3**, e28. (10.1017/ehs.2021.22) 37588564 PMC10427326

[40] Flache A, Mäs M, Feliciani T, Chattoe-Brown E, Deffuant G, Huet S, Lorenz J. 2017 Models of social influence: towards the next frontiers. J. Artif. Soc. Soc. Simul. **20**, 31. (10.18564/jasss.3521)

[41] Sobkowicz P. 2018 Opinion dynamics model based on cognitive biases of complex agents. J. Artif. Soc. Soc. Simul. **21**, 8. (10.18564/jasss.3867)

[42] Chen X, Zhang X, Xie Y, Li W. 2017 Opinion dynamics of social-similarity-based Hegselmann–Krause model. Complexity **2017**, 1-12. (10.1155/2017/1820257)

[43] Squazzoni F, Jager W, Edmonds B. 2014 Social simulation in the social sciences: a brief overview. Soc. Sci. Comput. Rev. **32**, 279-294. (10.1177/0894439313512975)

[44] Alizadeh M, Cioffi-Revilla C, Crooks A. 2015 The effect of in-group favoritism on the collective behavior of individuals’ opinions. Adv. Complex Syst. **18**, 1550002. (10.1142/S0219525915500022)

[45] Feliciani T, Flache A, Mäs M. 2021 Persuasion without polarization? Modelling persuasive argument communication in teams with strong faultlines. Comput. Math. Organ. Theory **27**, 61-92. (10.1007/s10588-020-09315-8)

[46] Sobkowicz P. 2020 Whither now, opinion modelers? Front. Phys. **8**, 461. (10.3389/fphy.2020.587009)

[47] Galesic M, Olsson H, Dalege J, van der Does T, Stein DL. 2021 Integrating social and cognitive aspects of belief dynamics: towards a unifying framework. J. R. Soc. Interface **18**, 20200857. (10.1098/rsif.2020.0857) 33726541 PMC8086875

[48] Festinger L. 1954 A theory of social comparison processes. Hum. Relat. **7**, 117-140. (10.1177/001872675400700202)

[49] Martins ACR. 2009 Bayesian updating rules in continuous opinion dynamics models. J. Stat. Mech.: Theory Exp. **2009**, P02017. (10.1088/1742-5468/2009/02/P02017)

[50] Gabriel N, O’Connor C. 2022 Can confirmation bias improve group learning? *MetaArXiv*. (10.31222/osf.io/dzych)

[51] Smaldino PE, Moser C, Pérez Velilla A, Werling M. 2023 Maintaining transient diversity is a general principle for improving collective problem solving. Perspect. Psychol. Sci. 17456916231180100. (10.1177/17456916231180100) 37369100 PMC10913329

[52] Steiglechner P. 2023 An opinion formation model with social identity and in-group bias. Zenodo.

[53] Druckman JN, Klar S, Krupnikov Y, Levendusky M, Ryan JB. 2021 Affective polarization, local contexts and public opinion in America. Nat. Hum. Behav. **5**, 28-38. (10.1038/s41562-020-01012-5) 33230283

[54] Karimi F, Génois M, Wagner C, Singer P, Strohmaier M. 2018 Homophily influences ranking of minorities in social networks. Sci. Rep. **8**, 11077. (10.1038/s41598-018-29405-7) 30038426 PMC6056555

[55] Watts DJ, Strogatz SH. 1998 Collective dynamics of ‘small-world’ networks. Nature **393**, 440-442. (10.1038/30918) 9623998

[56] Holland PW, Laskey KB, Leinhardt S. 1983 Stochastic blockmodels: first steps. Soc. Netw. **5**, 109-137. (10.1016/0378-8733(83)90021-7)

[57] Phillips LD, Edwards W. 1966 Conservatism in a simple probability inference task. J. Exp. Psychol. **72**, 346-354. (10.1037/h0023653) 5968681

[58] Acemoglu D, Ozdaglar A. 2011 Opinion dynamics and learning in social networks. Dyn. Games Appl. **1**, 3-49. (10.1007/s13235-010-0004-1)

[59] Geschke D, Lorenz J, Holtz P. 2019 The triple-filter bubble: using agent-based modelling to test a meta-theoretical framework for the emergence of filter bubbles and echo chambers. Brit. J. Soc. Psychol. **58**, 129-149. (10.1111/bjso.12286) 30311947 PMC6585863

[60] Flache A, Mäs M. 2008 Why do faultlines matter? A computational model of how strong demographic faultlines undermine team cohesion. Simul. Model. Pract. Theory **16**, 175-191. (10.1016/j.simpat.2007.11.020)

[61] Bramson A, Grim P, Singer DJ, Berger WJ, Sack G, Fisher S, Flocken C, Holman B. 2017 Understanding polarization: meanings, measures, and model evaluation. Philos. Sci. **84**, 115-159. (10.1086/688938)

[62] Turner MA, Smaldino PE. 2018 Paths to polarization: how extreme views, miscommunication, and random chance drive opinion dynamics. Complexity **2018**, e2740959. (10.1155/2018/2740959)

[63] Gestefeld M, Lorenz J, Henschel NT, Boehnke K. 2022 Decomposing attitude distributions to characterize attitude polarization in Europe. SN Soc. Sci. **2**, 110. (10.1007/s43545-022-00342-7)

[64] Epstein JM. 2008 Why model? *J. Art. Soc. Soc. Simul.* **11**, 4.

[65] Edmonds B et al. 2019 Different modelling purposes. J. Art. Soc. Soc. Simul. **22**, 6. (10.18564/jasss.3993)

[66] Smaldino PE. 2023 Modeling social behavior: mathematical and agent-based models of social dynamics and cultural evolution. Princeton, NJ: Princeton University Press.

[67] Wimsatt WC. 1987 False models as means to truer theories. In *Neutral models in biology* (eds M Nitecki, A Hoffman), pp. 23–55. Oxford, UK: Oxford University Press.

[68] Craver CF. 2006 When mechanistic models explain. Synthese **153**, 355-376. (10.1007/s11229-006-9097-x)

[69] Smaldino PE. 2017 Models are stupid, and we need more of them. In *Computational social psychology*, 1st edn (eds RR Vallacher, SJ Read, A Nowak), pp. 311–331. Frontiers of Social Psychology. New York, NY: Routledge.

[70] Kahan DM, Jenkins-Smith H, Braman D. 2011 Cultural cognition of scientific consensus. J. Risk Res. **14**, 147-174. (10.1080/13669877.2010.511246)

[71] Toomey AH. 2023 Why facts don’t change minds: insights from cognitive science for the improved communication of conservation research. Biol. Conservat. **278**, 109886. (10.1016/j.biocon.2022.109886)

[72] Mason L. 2015 ‘I disrespectfully agree’: the differential effects of partisan sorting on social and issue polarization. Am. J. Pol. Sci. **59**, 128-145. (10.1111/ajps.12089)

[73] Landrum AR, Lull RB, Akin H, Hasell A, Jamieson KH. 2017 Processing the papal encyclical through perceptual filters: Pope Francis, identity-protective cognition, and climate change concern. Cognition **166**, 1-12. (10.1016/j.cognition.2017.05.015) 28549233

[74] Cook J, Lewandowsky S. 2016 Rational irrationality: modeling climate change belief polarization using Bayesian networks. Top. Cogn. Sci. **8**, 160-179. (10.1111/tops.12186) 26749179

[75] Axelrod R. 1997 The dissemination of culture: a model with local convergence and global polarization. J. Conflict Resol. **41**, 203-226. (10.1177/0022002797041002001)

[76] Edmonds B. 2019 Using agent-based modelling to inform policy – what could possibly go wrong? In *Multi-agent-based simulation XIX* (eds P Davidsson, H Verhagen), vol. 11463, pp. 1–16. Cham, Switzerland: Springer International Publishing.

[77] Deffuant G, Neau D, Amblard F, Weisbuch G. 2000 Mixing beliefs among interacting agents. Adv. Complex Syst. **3**, 87-98. (10.1142/S0219525900000078)

[78] Hegselmann R, Krause U. 2002 Opinion dynamics and bounded confidence models, analysis and simulation. J. Art. Soc. Social Simul. **5**, 1-2.

[79] Mäs M, Flache A, Helbing D. 2010 Individualization as driving force of clustering phenomena in humans. PLoS Comput. Biol. **6**, e1000959. (10.1371/journal.pcbi.1000959) 20975937 PMC2958804

[80] Abelson RP. 1967 Mathematical models in social psychology. In *Advances in experimental social psychology* (ed. L Berkowitz), vol. 3, pp. 1–54. New York, NY: Academic Press.

[81] Boutyline A, Willer R. 2017 The social structure of political echo chambers: variation in ideological homophily in online networks. Polit. Psychol. **38**, 551-569. (10.1111/pops.12337)

[82] Cinelli M, Morales GDF, Galeazzi A, Quattrociocchi W, Starnini M. 2021 The echo chamber effect on social media. Proc. Natl Acad. Sci. USA **118**, e2023301118. (10.1073/pnas.2023301118) 33622786 PMC7936330

[83] Williams HTP, McMurray JR, Kurz T, Hugo Lambert F. 2015 Network analysis reveals open forums and echo chambers in social media discussions of climate change. Global Environ. Change **32**, 126-138. (10.1016/j.gloenvcha.2015.03.006)

[84] Jasny L, Waggle J, Fisher DR. 2015 An empirical examination of echo chambers in US climate policy networks. Nat. Clim. Change **5**, 782-786. (10.1038/nclimate2666)

[85] Lau DC, Murnighan JK. 1998 Demographic diversity and faultlines: the compositional dynamics of organizational groups. Acad. Manage. Rev. **23**, 325-340. (10.2307/259377)

[86] Johnson D, Levin S. 2009 The tragedy of cognition: psychological biases and environmental inaction. Curr. Sci. **97**, 1593-1603.

[87] O’Connor C, Weatherall JO. 2018 Scientific polarization. Eur. J. Philos. Sci. **8**, 855-875. (10.1007/s13194-018-0213-9)

[88] Swim JK, Aviste R, Lengieza ML, Fasano CJ. 2022 OK boomer: a decade of generational differences in feelings about climate change. Global Environ. Change **73**, 102479. (10.1016/j.gloenvcha.2022.102479)

[89] Fazelpour S, Steel D. 2022 Diversity, trust, and conformity: a simulation study. Philos. Sci. **89**, 209-231. (10.1017/psa.2021.25)

[90] Barkoczi D, Galesic M. 2016 Social learning strategies modify the effect of network structure on group performance. Nat. Commun. **7**, 13109. (10.1038/ncomms13109) 27713417 PMC5059778

[91] Clayton SD, Opotow S (eds). 2003 Identity and the natural environment: the psychological significance of nature. Cambridge, MA: MIT Press.

[92] Meleady R, Crisp RJ. 2017 Redefining climate change inaction as temporal intergroup bias: temporally adapted interventions for reducing prejudice may help elicit environmental protection. J. Environ. Psychol. **53**, 206-212. (10.1016/j.jenvp.2017.08.005)

[93] Ross AD, Rouse SM, Mobley W. 2019 Polarization of climate change beliefs: the role of the millennial generation identity. Soc. Sci. Quart. **100**, 2625-2640. (10.1111/ssqu.12640)

[94] Esposo SR, Hornsey MJ, Spoor JR. 2013 Shooting the messenger: outsiders critical of your group are rejected regardless of argument quality. Brit. J. Soc. Psychol. **52**, 386-395. (10.1111/bjso.12024) 23316747

[95] Hewstone M, Lolliot S, Swart H, Myers E, Voci A, Al Ramiah A, Cairns E. 2014 Intergroup contact and intergroup conflict. Peace Conflict: J. Peace Psychol. **20**, 39-53. (10.1037/a0035582)

[96] Brown R, Vivian J, Hewstone M. 1999 Changing attitudes through intergroup contact: the effects of group membership salience. Eur. J. Soc. Psychol. **29**, 741-764. (10.1002/(SICI)1099-0992(199908/09)29:5/6<741::AID-EJSP972>3.0.CO;2-8)

[97] Fielding KS, Hornsey MJ, Thai HA, Toh LL. 2020 Using ingroup messengers and ingroup values to promote climate change policy. Clim. Change **158**, 181-199. (10.1007/s10584-019-02561-z)

[98] Van Bavel JJ et al. 2020 Using social and behavioural science to support COVID-19 pandemic response. Nat. Hum. Behav. **4**, 460-471. 32355299 10.1038/s41562-020-0884-z

[99] Sabherwal A et al. 2021 The Greta Thunberg effect: familiarity with greta thunberg predicts intentions to engage in climate activism in the United States. J. Appl. Soc. Psychol. **51**, 321-333. (10.1111/jasp.12737)

[100] Masson T, Fritsche I. 2021 We need climate change mitigation and climate change mitigation needs the ‘We’: a state-of-the-art review of social identity effects motivating climate change action. Curr. Opin. Behav. Sci. **42**, 89-96. (10.1016/j.cobeha.2021.04.006)

[101] Marris E. 2019 Why young climate activists have captured the world’s attention. Nature **573**, 471-472. (10.1038/d41586-019-02696-0) 31551545

[102] Smaldino PE. 2020 How to translate a verbal theory into a formal model. Soc. Psychol. **51**, 207-218. (10.1027/1864-9335/a000425)

[103] Colleoni E, Rozza A, Arvidsson A. 2014 Echo chamber or public sphere? Predicting political orientation and measuring political homophily in Twitter using big data. J. Commun. **64**, 317-332. (10.1111/jcom.12084)

[104] Herdağdelen A, Zuo W, Gard-Murray A, Bar-Yam Y. 2013 An exploration of social identity: the geography and politics of news-sharing communities in Twitter. Complexity **19**, 10-20. (10.1002/cplx.21457)

[105] Bodrunova SS, Blekanov I, Smoliarova A, Litvinenko A. 2019 Beyond left and right: real-world political polarization in Twitter discussions on inter-ethnic conflicts. Media Commun. **7**, 119-132. (10.17645/mac.v7i3.1934)

[106] Steiglechner P, Smaldino PE, Moser D, Merico A. 2023 Social identity bias and communication network clustering interact to shape patterns of opinion dynamics. Figshare. (10.6084/m9.figshare.c.6960070) PMC10715916 38086404

